# High atmospheric dissolved organic nitrogen deposition in southeast Tibet

**DOI:** 10.1016/j.heliyon.2024.e39854

**Published:** 2024-10-28

**Authors:** Wei Wang, Xingyu Liu, Lixue Guan

**Affiliations:** a College of Resources and Environmental Sciences, Tibet Agriculture & Animal Husbandry University, Nyingchi, 860000, China; b Key Laboratory of Forest Ecology in Tibet, Ministry of Education, Tibet Agriculture & Animal Husbandry University, Nyingchi, Tibet, 860000, China

**Keywords:** Nitrogen deposition, Dissolved organic nitrogen, Southeast tibet, Different environment

## Abstract

Nitrogen deposition has been highlighted in the last decades because it was considered as one control factor of global change. The Yarlung Tsangpo Grand Canyon acts as a major passage of monsoonal moisture transport from the Bay of Bengal into the Tibetan Plateau. However, the characteristics of nitrogen(N) deposition in this area are still unclear. Here, we established five N deposition monitoring sites and quantification the bulk N deposition fluxes from 2200 to 4600m above sea level in southeast Tibet. Results showed that the average precipitation amount of the five sites was 1127.7 mm. The dissolved organic nitrogen (DON) was the dominant species, and the deposition flux was 16.80 kg N ha^−1^ yr^−1^. The averaged NH_4_^+^-N deposition flux was 4.92 kg N ha^−1^ yr^−1^, whereas the NO_3_^−^-N deposition flux was 1.49 kg N ha^−1^ yr^−1^. In addition, the deposition fluxes of TDN, DON and NH_4_^+^-N were all significantly positive to precipitation amounts at all five sampling sites. However, the deposition flux of NO_3_^−^-N was significantly correlated with precipitation amount in the remote environment, and there was no correlation between precipitation amounts and NO_3_^−^-N deposition fluxes in human concentrated areas. TDN, DON and NH_4_^+^-N deposition were all concentrated in the plant growing season at all five sampling sites. In conclusion, the atmospheric TDN deposition flux in the Yarlung Zangbo Grand Canyon in southeast Tibet is mainly controlled by precipitation, and DON was the dominant species, followed by NH_4_^+^-N, and NO_3_^−^-N contribution was limited.

## Highlights

• Precipitation N deposition were studied in areas of The Yarlung Tsangpo Grand Canyon.
• DON was the dominant species, followed by NH_4_^+^-N.
• The deposition fluxes of TDN, DON were controlled by precipitation amount.
• Both concentration and deposition flux of NO_3_^−^-N was higher in human concentrated area.

## 1 Introduction

In the past few decades, atmospheric nitrogen(N) pollution caused by intensified anthropogenic activities has increased significantly, which in turn has led to a significant increase in atmospheric N deposition flux [[1], [2], [3], [4]]. N is the most important nutrient element of organisms in the ecosystem [5], and the increase of environmental N input will undoubtedly change the N cycle of the local ecosystem to a certain extent, which is manifested in the promotion of ecosystem productivity when it is below the critical threshold, and the negative effects on the ecosystem when it is above the critical threshold, such as soil acidification and loss of plant diversity [[6], [7], [8]]. The source, composition, and transformation of dissolved organic nitrogen (DON) in the atmosphere are complex and difficult to quantify [9], while the impact of inorganic N on organisms and the environment is more effective, so the quantitative study of atmospheric inorganic N has arisen more attention, while the studies focused on DON deposition have received rare attention [[3], [6], [10]]. At the global or national scale, it has been reported that DON deposition accounts for about one-third of the total dissolved nitrogen (TDN) deposition at large scales [[11], [12], [13]]. However, the contribution of DON to TDN fluxes may be up to 90 percent at some sampling sites [11]. It has been reported that DON is also taken up by plants, especially in N-limited ecosystems [14], so the quantification of DON flux should be given more importance in nitrogen-limited ecosystems.

Atmospheric DON can be roughly divided into reduced organic nitrogen, oxidized organic nitrogen and biological organic nitrogen. Biological organic sometimes includes reduced and oxidized organic nitrogen as large particles [9]. In general, DON can be divided into natural sources and anthropogenic sources, that is, natural sources are also the main sources of atmospheric DON, which means, there may not be a direct linear relationship between the deposition flux of atmospheric DON and the intensity of anthropogenic activities [9,11,15]. Conversely, in the environment with low anthropogenic disturbance with complex vegetation, the concentration of inorganic N in the atmosphere may also be at a low level due to the low emission from anthropogenic sources, which may result in low inorganic N concentration in precipitation, and high DON percentage of atmospheric N deposition in those areas was observed [13].

In southeastern Tibet, where anthropogenic activities are low, the vegetation in this region is extremely rich under the influence of warm and humid air currents from the Indian Ocean, especially the forest stock volume is much higher than other areas at the national scale [16]. In addition, the source of the air mass in the Yarlung Tsangpo Grand Canyon is the Indian Ocean [17], which will climb rapidly and cause heavy precipitation in a very short horizontal distance, which may lead to a certain degree of enrichment of atmospheric pollutants in this region, especially in precipitation, but so far there are limited report on the quantification of atmospheric N deposition in this region, especially the quantitative study of DON deposition. Therefore, in this study, atmospheric N deposition monitoring sites were set up at different altitudes in southeast Tibet under different vegetation background conditions to clarify 1) quantification the bulk TDN deposition flux in southeast Tibet; 2) understand the dominant TDN species of TDN deposition in this area; 3) Are the flux and control factors of different N species comparable at different environment.

## 2 Materials and methods

### 2.1 Location description

**Fig. 1:**
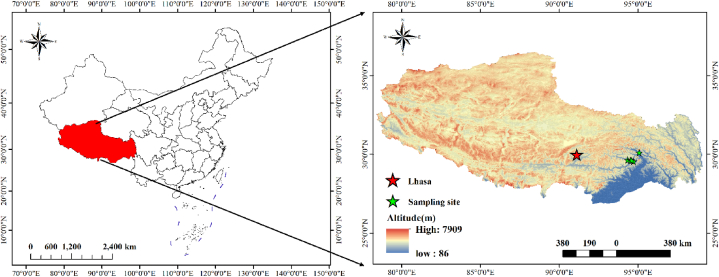
Sampling location.

The study area is mainly in the area of the Yarlung Zangbo water vapor channel in southeast Tibet (Fig. 1), where the warm and humid airflow of the Indian Ocean brings abundant rainfall and heat along the Yarlung Zangbo River Grand Canyon to the north, forming different climate zones such as tropical, subtropical, temperate, plateau temperate and plateau subarctic with the altitude increasing. In this study, N deposition sampling sites were set up according to the differences in natural vegetation, including mixed coniferous and broad-leaved forest area (TM), dark coniferous forest area (SJL1), shrub meadow area (SJL2), and meadow area (SJL3). At the same time, the sampling site was set up in Nyingchi City (LZ), and the surrounding vegetation was the coniferous forest. It should be pointed out, the TM sampling site is located in a village, and the LZ sampling site is at the edge of Nyingchi City, which may be disturbed by anthropogenic emission sources to a certain extent. The sampling sites of SJL1, SJL2 and SJL3 are all sampling sites, and the only pollution sources may be G318 National Highway.

### 2.2 Sampling methods

Precipitation was collected across previous studies [18]. In short, atmospheric precipitation samples were collected using rain gauges (Central Tianyi Meteorological Instrument Co., Ltd., Tianjin, China). Rainwater samples were collected from five sites in southeastern Tibet from December 1, 2020 to December 1, 2022. When collecting, the rainfall is measured with a special measuring cylinder and the rainfall is recorded; The mixed precipitation samples were then packed into cleaned polyethylene bottles (100 ml), brought back to the laboratory, filtered by a 0.45 μm membrane, and refrigerated for analysis. The sampling time of TM sampling sites is from June 1, 2021, to August 1, 2022. The LZ and TM sampling sites were collected after rain, and the sampling numbers was 65 and 109, respectively. SJL1, SJL2, and SJL3 were sampled once a week, and the number of samples was 47, 30, and 32, respectively. It should be pointed out that precipitations were collected by a permanently open funnel system in this work. This means what we collected is bulk deposition which encompasses both wet and a portion of dry deposition.

### 2.3 Analysis methods

Firstly, TN was digested to NO_3_^−^-N for measurement. The digestive method was carried out according to the National Standard HJ 636–2012. Briefly, alkaline potassium persulfate is used to convert the nitrogenous compounds in the sample into nitrate under high temperature and high-pressure environments. The specific laboratory test steps are as follows: take 5 ml of sample and add it to a 25 ml graduated colorimetric tube with a plug grinding mouth, add 5 ml of alkaline potassium persulfate respectively, cover the plug and wrap it with gauze, put it into an autoclave, adjust the temperature and time to 120 °C and 30 min, open the valve to release gas, wait for natural cooling, take out the colorimetric tube, and cool to room temperature. 1 ml of HCl solution was added to each colorimetric tube, and the volume was fixed to 25 ml of the reticle and then shaken well for testing. The determination method is the same as that of NO_3_^−^-N.

Ammonium nitrogen (NH_4_^+^-N) and nitrate nitrogen (NO_3_^−^-N) in rainwater samples were determined by Smart Chem 450 automatic interrupted chemical analyzer (SmartChem 450, AMS Alliance, Italy). NO_3_^−^-N was reduced to NO_2_^−^-N and then determined, so the NO_3_^−^-N measured in this study included nitrate and nitrite.

### 2.4 Calculation method

The concentrations of NH_4_^+^-N and NO_3_^−^-N were directly determined for all samples. However, the DON concentration in each sample was calculated as follows:

$$C_{\text{DON}}=C_{\text{TN}}-{C_{\text{NH}4}^{+}}_{-N}-{C_{\text{NO}3}^{-}}_{-N}$$

Volume-weighted mean concentrations (VWM) were used for comparison in this study, which were calculated as follow:

First, the deposition fluxes of different N form were calculated as follow:

Depi=Pi∗Ci/100

Depi means deposition fluxes of a N species in the ith sample (kg ha^−1^), Pi is the precipitation amount collected within the ith sampling period(mm), Ci is the concentration of corresponding N species in the ith sample. 100 is the unit conversion factor (mg L^−1^ to kg ha^−1^).

Second, deposition fluxes of different N species were calculated as follow:

$$F=\sum_{1}^{i}Dep\;i$$

$$P=\sum_{1}^{i}P\;i$$

F represents the seasonal or annual bulk deposition fluxes of a N species (kg ha^−1^); P represents the annual precipitation amounts.

Third, the VWM concentration of an ion is calculated using the following formula:

C=100×F/P

F means the bulk deposition fluxes, P means the precipitation, and 100 is the conversion factor of kg N ha^−1^ L^−1^ to mg L^−1^.

In addition, March ∼ May is defined as spring, June–August is defined as summer, September ∼ November is defined as autumn, and December to February is defined as winter.

### 2.5 Data analysis methods

Origin2024 was used for graph analysis.

## 3 Results

### 3.1 Comparison of annual precipitation, N deposition and VWM concentration

**Fig. 2:**
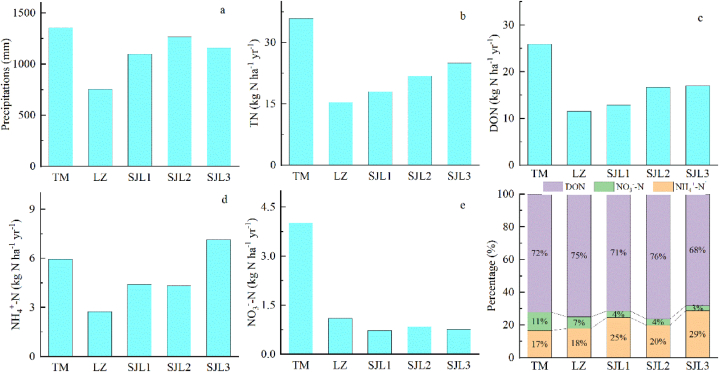
Comparison of annual precipitation amount, N deposition flux at five sampling sites.

**Fig. 3:**
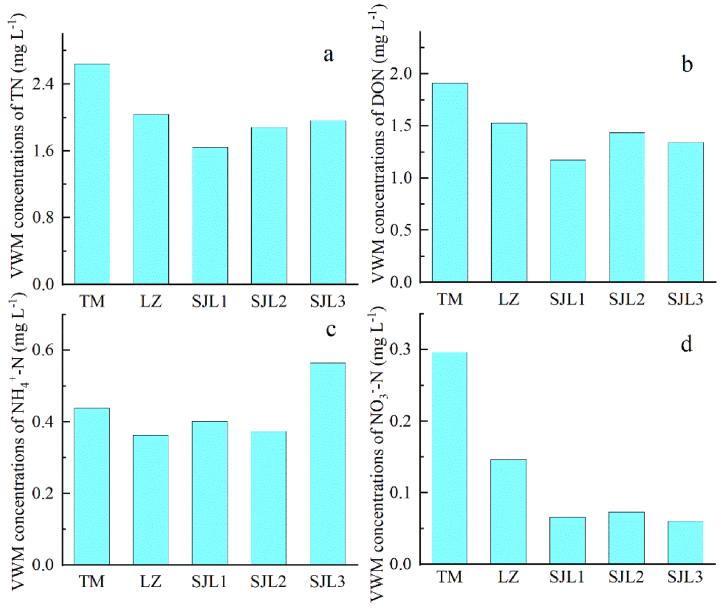
Comparison of VWM concentrations of different N species at five sampling sites.

The annual precipitation ratios of TM, LZ, SJL1, SJL2 and SJL3 were 1357.6, 755.3, 1099.0, 1267.2 and 1159.3 mm (Fig. 2a). Obviously, the precipitation amount was highest in TM sampling site and lowest in LZ sampling site. Corresponding to precipitation amount, the TN deposition fluxes at different sampling sites were 35.9, 15.40, 18.0, 21.8 and 24.9 kg N ha^−1^ yr^−1^ (Fig. 2b), respectively, which was the highest in TM and the lowest in LZ sampling sites. The deposition flux of DON showed similar patterns, with the highest value of 25.9 kg N ha^−1^ yr^−1^ in TM and the lowest value of 11.6 kg N ha^−1^ yr^−1^ in LZ (Fig. 2c). The deposition flux of NH_4_^+^-N was 5.95, 2.74, 4.42, 4.33 and 7.16 kg N ha^−1^ yr^−1^ at TM, LZ, SJL1, SJL2 and SJL3 sampling sites (Fig. 2d), respectively, with the highest value at SJL3 and the lowest value at LZ. The NO_3_^−^-N deposition flux was 4.02, 1.10, 0.73, 0.84 and 0.76 kg N ha^−1^ at TM, LZ, SJL1, SJL2 and SJL3 sampling sites (Fig. 2e), respectively, where the highest value was observed in TM, and the lowest value was observed in SJL1. Overall, the average precipitation amounts of the five sites was 1127.7 mm, the average TN deposition was 23.2 kg N ha^−1^ yr^−1^, whereas the averaged NH_4_^+^-N deposition flux was 4.92 kg N ha^−1^ yr^−1^, and NO_3_^−^-N deposition flux was 1.49 kg N ha^−1^ yr^−1^, and DON deposition flux was 16.80 kg N ha^−1^ yr^−1^. Noticeably, DON is the dominant form of N deposition in the region. The proportion of DON to DTN was lowest in SJL3, with values of 68.2 %, and highest in SJL1, with values of 76.3 % (Fig. 1f). Among the five sampling sites, VWM concentration of DTN was highest at TM, with value of 2.64 mg N L^−1^, and lowest at SJL, with value 1.64 mg N L^−1^ (Fig. 3a). VWM concentration of DON was highest at TM, with value 1.91 mg N L^−1^, and lowest in SJL1, with value 1.17 mg N L^−1^(Fig. 3b); VWM concentration of NH_4_^+^-N was highest in SJL3 (0.57 mg N L^−1^), and lowest in LZ (0.36 mg N L^−1^) (Fig. 3c). VWM concentration of NO_3_^−^-N was highest in TM (0.30 mg N L^−1^) and lowest in SJL3(0.06 mg N L^−1^) (Fig. 3d).

### 3.2 Seasonal variations of N deposition fluxes and VWM concentrations

**Fig. 4:**
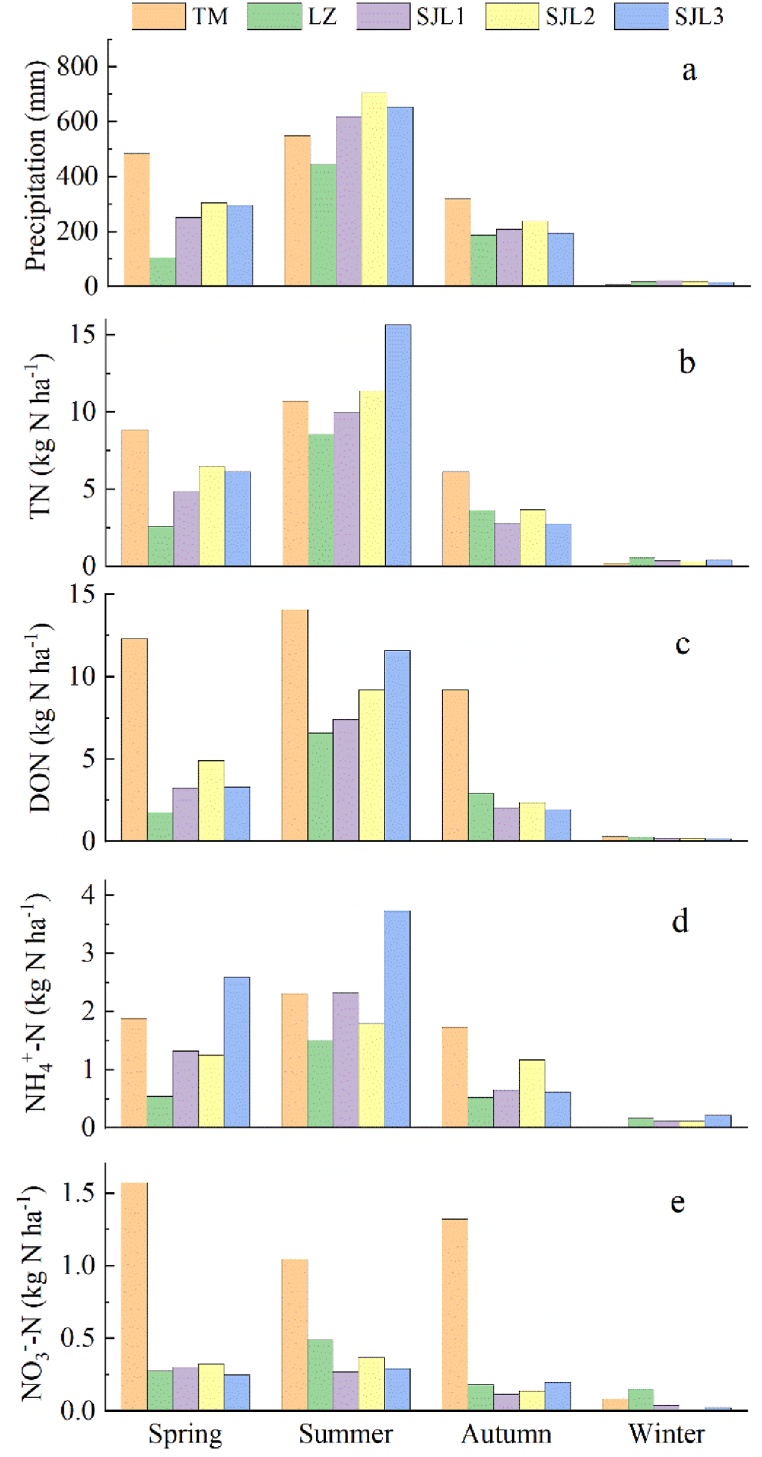
Seasonal variations of N deposition flux at five sampling sites.

The precipitation amount showed similar patterns at the five sampling sites, which were highest in summer and lowest in winter. As a result, TN, DON and NH_4_^+^-N deposition fluxes were higher in summer than those values in other seasons at all five sampling sites. However, NO_3_^−^-N deposition flux was highest in spring at the TM sampling site (Fig. 4).

**Fig. 5:**
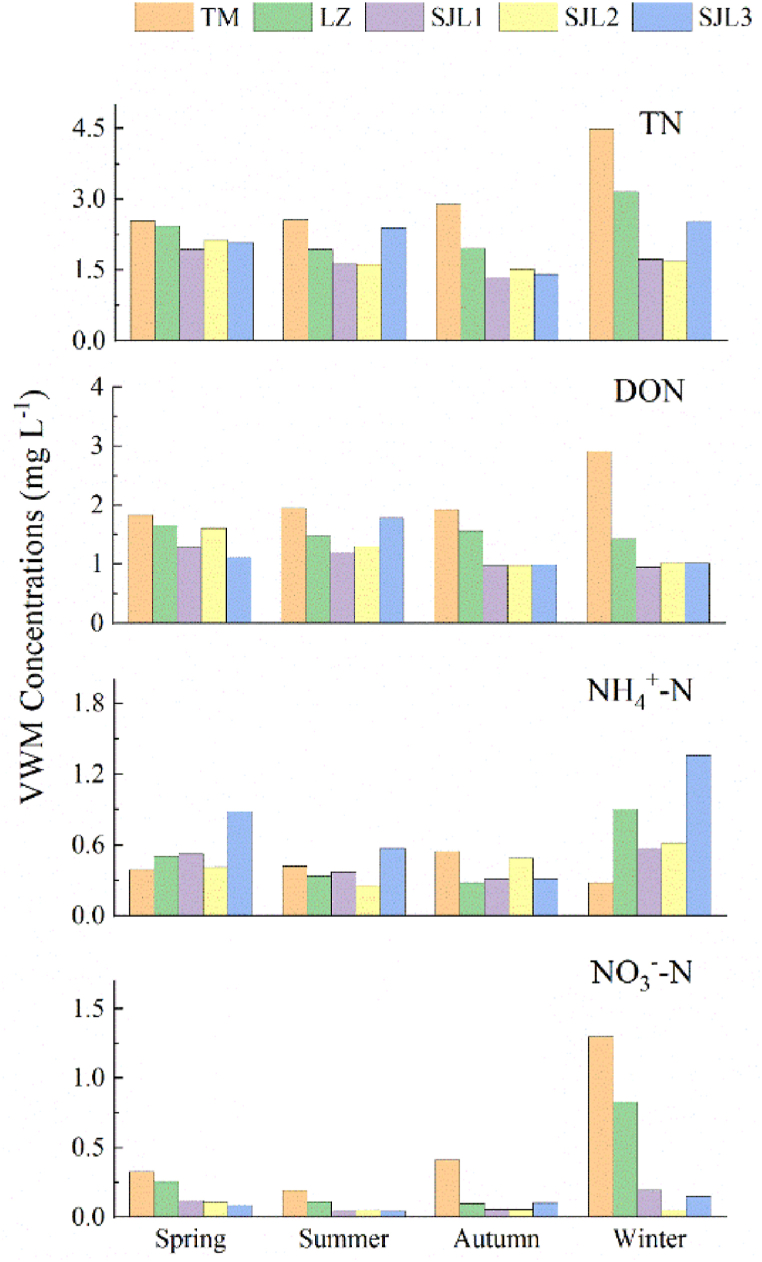
Seasonal variations of VWM concentration at five sampling sites.

Seasonal variation of the DON VWM concentrations were shown as a pattern of TM > LZ ≈ SJL2>SJL1> SJL3 in spring, whereas shown the pattern of TM > SJL3> LZ > SJL2> SJL1 in summer, and TM > LZ > SJL3≈SJL2≈SJL1 in autumn at the five sampling sites (Fig. 5). Seasonal variation of the NH4+-N VWM concentrations were shown a pattern of SJL3>SJL1>LZ > SJL2>TM in spring, whereas shown the pattern of SJL3>TM > SJL1>LZ > SJL2 in summer, and TM > SJL2 >SJL3≈SJL1>LZ in autumn at the five sampling sites. In addition, The VWM concentrations of DON were all highest in TM sampling sites in all seasons (Fig. 5).

### 3.3 Linear fit between precipitation amounts and N deposition fluxes

**Fig. 6:**
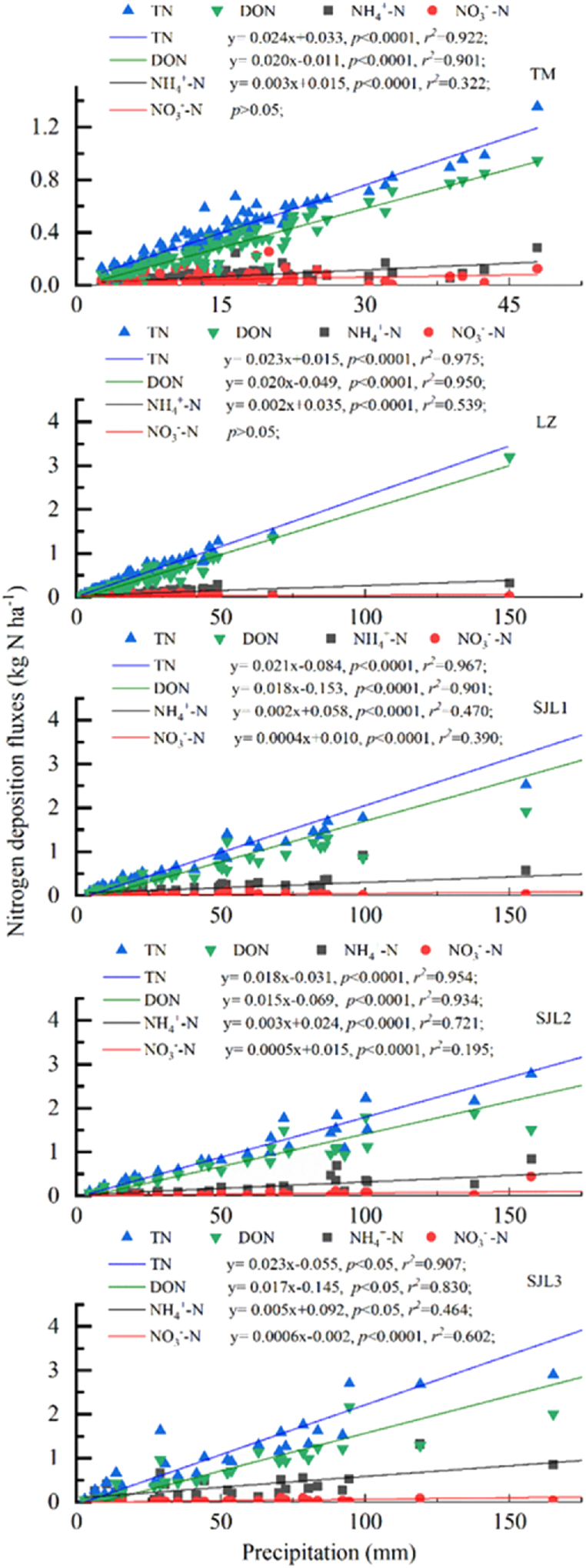
Liner fitting between N deposition flux and precipitation amount.

The linear fitting results revealed that a significant correlation between precipitation amounts and TN deposition fluxes, precipitation amounts and DON deposition fluxes, precipitation amounts and NH_4_^+^-N deposition fluxes existed at the five sampling sites (p < 0.0001). NO_3_^−^-N was significantly correlated with precipitation amount at SJL1, SJL2 and SJL3 sampling sites, but there was no correlation at LZ and TM sites (Fig. 6).

## 4 Discussion

### 4.1 Comparison of DON concentrations or deposition fluxes

DON is an important component of atmospheric N species, which plays an important role in N deposition quantification in terrestrial ecosystems [9]. Based on previous studies, Cornell et al. showed that the concentration of DON in precipitation ranged from 1.8 to 89 μmol N L^−1^, and the percentage of DON to TDN ranged from 5 to 88 % [11]. At regional scale, it had been reported that the concentration of DON in rainwater ranged from 0.15 to 1.74 μmol N L^−1^, accounting for 2 %–38 % of TDN concentrations [15]. In the same period, the VWM conentrations of DON were reported 13–190 μmol N L^−1^, and the percentage of DON to TDN ranged from 7 % to 67 % [13]. Recently, DON deposition flux in rainwater in China was 4.32 kg N ha^−1^, accounting for 30.3 percent of the TDN deposition [12]. to further understand the situation of DON deposition in our research area, DON concentrations/deposition fluxes reported in similar environments or at large scales were summarized in Table 1. We found that both DON concentrations and deposition fluxes in southeast Tibet were much higher than those in other areas in precipitation. DON concentration in our research area was comparable to the study in the North China Plain (101 mmol L^−1^), where there are high agriculture or no agriculture pollution sources.

**Table 1:** DON Concentrations or deposition fluxes in similar environment or large scale.

Sampling Area	DON Concentrations (μmol N L^−1^)	DON Deposition flux (kg N ha^−1^ yr^−1^)	DON/DTN	References
South west China		6.63	41.8 %	[12]
Qinghai—Tibet		4.52	35.9 %	[12]
Qinghai—Tibet	30		50.0 %	[19]
Dashanchong, China	12.9		11.1 %	[20]
Yamanashi, Japan		6.10	10.4 %	[21]
Uljin, Korea		2.66	28.0 %	[22]
Puerto Rico		0.98	41.0 %	[23]
Atlantic Forest reserve, Brazil	10.1		26.6 %	[24]
Atlantic Forest reserve, Brazil	10.9		32.0 %	[24]
Northeast China	5.86	4.45	23.0 %	[25]
China	77	6.84		[13]
Eastern Mediterranean	4.8	1.96	22.7 %	[26]
Continental	21		36 %	[11]
Compiled Data	21.4	3.1	34 %	[9]
Southeast Tibet	105.5	16.8	72.6 %	This Study

In the Yarlung Zangbo Grand Canyon located in southeast Tibet, there are different climates and vegetation types from tropical rainforest to alpine ice and snow zone within a horizontal distance of more than 150 km, and plant species are extremely rich. On one hand, plants and atmospheric bacteria are extremely important sources of atmospheric organic nitrogen [9], and plant species are extremely abundant on the vertical gradient of 5000m above sea level in the study area, which provides a guarantee for the source of organic nitrogen. On the other hand, the precipitation in the study area in the rainy season was mostly caused by cloud and fog events in the canyon, rather than a single long-distance air mass transport, and the organic nitrogen content (e.g. Dissolved free amino acids, Total hydrolyzable amino acids) in the clouds and fog was extremely high [11]. In addition, the precipitation in this region is dominated by the moisture transport over the Yarlung Tsangpo Grand Canyon water vapor channel, which means the Ganges Delta-the world's largest delta was located in the airmasses sources direction [17,27]. The Ganges Delta has fertile soil and developed agriculture, which pollution from agricultural sources may make the air masses rich in NH_4_^+^-N and DON from this direction source. When the air mass from the south direction is transported up the Yarlung Tsangpo River, the water vapor channel gradually narrows due to the topography, which may lead to pollutants enrichment in the atmosphere. In addition, the altitudes of the sampling sites are relatively high, and the high-level water vapor transport in the Yarlung Zangbo Grand Canyon is more likely to affect the precipitation in the eastern hinterland of the Tibetan Plateau [17]. As a result, DON concentration was at a high level and showed consistent regularity in all 5 sampling sites.

### 4.2 Seasonal variations in atmospheric N deposition

Atmospheric N deposition flux showed significant seasonal variations [18,28,29]. The atmospheric N deposition fluxes are directly related to the N concentration in rainwater and precipitation amount [30]. Similar results were observed in this work (Fig. 6), and high deposition fluxes of different N species occurred in summer which has a high precipitation amount (Fig. 4). In addition, the *r*^*2*^ of linear fit between precipitation amount and DON deposition flux was at extremely high levels in all 5 sampling sites, that means, DON deposition flux was dominated by precipitation amount. This result indicated that long-distance transports were the predominant factors that controlled DON concentrations in precipitation (Fig. 6), and below-cloud scavenging showed limited contribution. The concentration of NH_4_^+^-N showed a significant positive correlation with precipitation amounts, but the values of *r*^*2*^ showed large variations at different sampling sites (Fig. 6), which revealed that NH_4_^+^-N pollution sources were different in different sampling sites or different sampling periods. NO_3_^−^-N deposition fluxes were positively related to precipitation amounts in remote environments but no liner correlation was observed in LZ, TM sampling sites (Fig. 6), suggesting that NO_3_^−^-N sources may be largely influenced by seasonal variation of local pollution emission sources in LZ, TM sampling sites. In addition, a decrease in atmospheric NO_2_ concentrations has been reported as a result of lockdown measures to reduce the spread of COVID-19 [[31], [32], [33]]. Traffic pollution is the main NO_2_ emission source in LZ city and transportation was almost completely prohibited from August and November 2022 because of COVID-19. Here, precipitation samples collected from August and November 2022 were selected as one group, and the other precipitation samples were classified into another group. Results showed that the NO_3_^−^-N concentration averaged 0.05 mg N L^−1^ for the former and 0.22 mg N L^−1^ for the latter. Obviously, the COVID-19 lockdowns significantly reduced the atmospheric NO_3_^−^-N deposition fluxes in LZ city.

In general, the nitrogen concentration in rainwater is negatively correlated with precipitation [34]. That is, the higher the precipitation, the lower the concentration of N in the precipitation [3,35]. This study showed that NH_4_^+^-N and NO_3_^−^-N showed higher N concentrations in winter when precipitation was extremely low, but DON did not show similar patterns (Fig. 5). The reason might be due to the fact that the source direction of the winter air mass in the study area was not southern, and it was not the plant growing season except for the TM sampling site may lead to lack of DON sources. In summer, On the one hand, the precipitation in the study area was mainly dominated by air masses from the south [17], and on the other hand, pollen produced during the growing season was an important source of atmospheric DON.

### 4.3 Differences of N deposition flux among the five sampling sites

The precipitation amount and the precipitation fluxes of TN, DON and NO_3_^−^-N in the rainfall water were the highest at the TM sampling site (Fig. 2). On one hand precipitation amount showed a high level in Spring and Autumn at TM sampling site (Fig. 4). In the other hand, the concentrations of DON and NO_3_^−^-N at this sampling site were at a high level (Fig. 3), which may be due to the fact that the surrounding environment is a village, and there are certain anthropogenic emission sources, such as biomass combustion. In addition, this sampling site is located at the bottom of the canyon, which may not be conducive to atmospheric pollutant diffusion. Except for the TM and SJL3 sampling site, the TN and DON deposition fluxes were all showed an increasing trend with altitude increasing (Fig. 2). These results can partially be explained by the increased precipitation amount in different altitudes (Fig. 6). At SJL3 sampling site, high DON and NH_4_^+^-N were observed in summer, the possible reason might be that the freezing and thawing effect is frequently and severely from April to June in SJL3 sampling sites, which may results plant and animal residues that rich in N and covered by snow in winter decomposed intensively. Then, NH_3_ and DON might be emitted into the atmosphere in this process, but the cold and humid ambient temperature makes the atmospheric NH_3_ and DON not easy to diffuse, in contrast, atmospheric NH_3_ and DON are easily scavenged by the condensation water in the ambient environment and then back to the ground by precipitation events. Meanwhile, the NH_3_ was easily transported to the mountains during the upslope event [36], the atmospheric temperature will gradually decrease with the increase of altitude, and precipitation will be formed by the scavenging of condensed cloud water droplets and other pollutants at high altitudes, so the NH_4_^+^ concentration at SJL3 sampling site which located as high as 4600m (a.s.l.) is high. It is no doubt that the temperature is high in summer and NH_3_ can reach higher altitudes in summer in our study area. Specifically, in the three remote sampling sites, it can be seen that the NO_3_^−^-N concentration in SJL2 was slightly higher than that in the other two sites (Fig. 2). the reason might be that SJL2 sampling sites might be influenced by the automobile exhaust to some extent from G318 National Highway, when precipitation events occur in this area in spring, the road covered by snow and ice often causes traffic jams, that is, the increase of automobile exhaust emissions caused by traffic jams makes the atmospheric NO_3_^−^-N concentration increased. At the same time, due to the relatively low NO_3_^−^-N concentration in precipitation in the study area, the occurrence of these accidental events may contribute to the increases of NO_3_^−^-N increasing.

### 4.4 Implications

Previously, studies have shown that plants tend to absorb ammonia nitrogen in temperate regions [37]. In coniferous forests on the Tibetan Plateau or in boreal regions, organic nitrogen is the most important nitrogen source for plants [14,38]. In the southeastern Tibet region, the forest ecosystem has the highest carbon density in China [16]. It is also reported a large group of high trees, including the tallest tree in Asia. Plants absorb and utilize organic nitrogen and ammonia nitrogen, which undoubtedly consumes less energy [39]. So, are the environmental N input forms dominated by organic nitrogen and ammonia nitrogen in southeast Tibet of great significance for the formation and growth of vegetation in this region? That is, the preference of dominant plants in the study area to use nitrogen forms deserves further study.

## 5 Conclusions

In this study, we measured atmospheric nitrogen deposition fluxes in the Yarlung Zangbo Grand Canyon area for two years. The annual deposition fluxes of TN, NH_4_^+^-N, NO_3_^−^-N and DON averaged 23.2, 4.92, 1.49 and 16.8 kg N ha^−1^year^−1^, respectively. DON contributed 72.4 % to TN, suggesting the importance of DON as a contributor to N deposition. In addition, the deposition fluxes of TN, NH_4_^+^-N and DON were mainly affected by precipitation on both spatial and temporal scales. Although the contribution of NO_3_^−^-N to TN is lower, higher VWM concentrations of NO_3_^−^-N in human-concentrated areas indicate that local emissions dominate NO_3_^−^-N deposition fluxes in southeast Tibet. Our results suggested that ignoring the contribution of organic nitrogen deposition will cause great uncertainty in the quantification of atmospheric nitrogen deposition and ecological nutrient cycling in southeastern Tibet.

## CRediT authorship contribution statement

**Wei Wang:** Writing – original draft, Visualization, Validation, Supervision, Software, Resources, Project administration, Methodology, Investigation, Funding acquisition, Formal analysis, Data curation, Conceptualization. **Xingyu Liu:** Software. **Lixue Guan:** Investigation, Formal analysis.

## Data availability

Data will be made available on request.

## Funding

This work was supported by the 10.13039/501100001809National Natural Science Foundation of China (42067036).

## Declaration of Competing Interest

The authors declare that they have no known competing financial interests or personal relationships that could have appeared to influence the work reported in this paper.
